# Three-Dimensional Skyrmions with Arbitrary Topological Number in a Ferromagnetic Spin-1 Bose-Einstein Condensate

**DOI:** 10.1038/s41598-019-54856-x

**Published:** 2019-12-11

**Authors:** Huan-Bo Luo, Lu Li, Wu-Ming Liu

**Affiliations:** 1 Institute of Theoretical Physics, State Key Laboratory of Quantum Optics and Quantum Optics Devices, Shanxi University, Taiyuan, 030006 China; 2 Beijing National Laboratory for Condensed Matter Physics, Institute of Physics, Chinese Academy of Sciences, Beijing, 100190 China

**Keywords:** Ultracold gases, Matter waves and particle beams

## Abstract

We propose a new scheme for creating three-dimensional Skyrmions in a ferromagnetic spin-1 Bose-Einstein condensate by manipulating a multipole magnetic field and a pair of counter-propagating laser beams. The result shows that a three-dimensional Skyrmion with topological number *Q* = 2 can be created by a sextupole magnetic field and the laser beams. Meanwhile, the vortex ring and knot structure in the Skyrmion are found. The topological number can be calculated analytically in our model, which implies that the method can be extended to create Skyrmions with arbitrary topological number. As the examples, three-dimensional Skyrmions with *Q* = 3, 4 are also demonstrated and are distinguishable by the density distributions with a specific quantization axis. These topological objects have the potential to be realized in ferromagnetic spin-1 Bose-Einstein condensates experimentally.

## Introduction

Skyrmions are topological objects that were first proposed in high-energy physics in 1960s^1^, and nowadays attract much attention in many condensed matter systems, such as superconductivity^2^, quantum Hall systems^3^, liquid crystals^4^, magnetic systems^5^, and Bose-Einstein condensates (BECs). The order parameters of BECs have rich structures due to the spin degree of freedom and can support various topological objects, e.g. solitons^6,7^, vortices^8–10^, monopoles^11–15^ and knots^16,17^. With the development of magnetic and optical techniques, one can manipulate precisely the spin in BECs. Thus, BEC system provides an ideal platform to study the dynamics of topological spin structures, such as Skyrmions.

In BEC systems, two-dimensional Skyrmions have been studied massively in theory^18–20^ and realized in experiment by using the phase-imprinting technique^21^. In general, three-dimensional Skyrmions are unstable toward shrinkage due to the gradient energy. There are two strategies to realize a three-dimensional Skyrmion in a BEC. The first is to introduce a non-Abelian gauge field^22^, which will prevent the Skyrmion from shrinkage so that a stable Skyrmion can be formed in a ground state of the BEC. Another is the dynamical creation of three-dimensional Skyrmion and then study it at a short time before it shrinks^23^. Recently, using the later strategy, a three-dimensional Skyrmion (Shankar Skyrmion) with unit topological number was experimentally realized in a ferromagnetic spin-1 BEC by manipulating a three-dimensional quadrupole magnetic field^24^. In addition, a recent study also described the creation of 3D skyrmions in the cyclic and nematic phases of spin-2 BEC by solving the 3D Gross-Pitaevskii equation^25^. In the spin-1 ferromagnetic BEC, order parameter manifold has the *SO*(3) symmetry and can host a topological object known as a Shankar Skyrmion^26^. The Skyrmion can be identified by estimating its topological number. However, due to the lack of suitable magnetic field, the Shankar Skyrmions with higher topological number (*Q* ≥ 2) have not yet been well studied.

In this paper, we propose a new scheme for creating a three-dimensional Skyrmion in a ferromagnetic spin-1 BEC by manipulating a multipole magnetic field and a pair of counter-propagating laser beams. Thus, atomic spin is affected by the light field and behaves like it in the magnetic field. At first, we present a model and the new results are obtained by means of numerical simulations based on this model. We simulate their dynamical creation of Skyrmion with *Q* = 2 and compare the topological properties of the Skyrmions with *Q* = 2, 3, 4. We also present analytic results to support our discussion. All the results suggest that it is possible to create and observe Shankar Skyrmions with higher topological number in experiments.

## Results

### Model

We consider a typical ^87^Rb BEC trapped in an optical potential *V*(**r**). The dynamics of the BEC at an external magnetic field **B**(**r**) can be described by the three-dimensional spin-1 Gross-Pitaevskii (GP) equation

1 $$i\hslash {{\rm{\partial }}}_{t}\Psi =[-\frac{{\hslash }^{2}{{\rm{\nabla }}}^{2}}{2m}+V+{c}_{0}n+({\mu }_{B}{g}_{F}{\bf{B}}+{c}_{2}n{\bf{S}})\cdot {\bf{F}}]\,\Psi ,$$

where $\Psi =\sqrt{n}\xi$ is the order parameter with *n* being the atomic density and *ξ* = (*ξ*_1_,*ξ*_0_,*ξ*_−1_)^*T*^ being the three-component spinor. *c*_0_ = 4*π*$\hslash$^2^(*a*_0_ + 2*a*_2_)/(3 *m*) and *c*_2_ = 4*π*$\hslash$^2^(*a*_2_ − *a*_0_)/(3 *m*) are density-density and spin-spin coupling constants with *a*_0_ = 5.387 nm, *a*_2_ = 5.313 nm, and *m* = 1.443 × 10^−25^ kg for ^87^Rb^27,28^. In this case, *c*_2_ < 0, which implies that the ground state is ferromagnetic in the absence of external magnetic field^29^. The total number of particles is chosen to be *N* = 4.4 × 10^4^. *g*_*F*_ = −1/2 is the Landé g factor for ^87^Rb and *μ*_*B*_ is the Bohr magneton. The optical trapping potential has the form of *V*(**r**) = *mω*^2^(*x*^2^ + *y*^2^ + *z*^2^)/2 with *ω* = 100 Hz being optical trapping frequency. **F** = (*F*_*x*_, *F*_*y*_, *F*_*z*_)^*T*^ is a vector of spin-1 Pauli matrices and the local spin is ${\bf{S}}({\bf{r}},t)={\xi }^{\dagger }{\bf{F}}\xi$. In our model, we use weak magnetic field (about 100 G) and the evolution time is short (within 1 ms), thus linear Zeeman effect is dominant and the quadratic Zeeman effect is omitted. In addition, we do not consider the effect of Earth magnetic field, because we can always add a uniform basis magnetic field in z-axis to balance the Earth magnetic field in real experiment.

The general form of the spinor for the ferromagnetic phase can be obtained by rotating the standard spinor *ξ*_0_ = (1, 0, 0)^*T*^ as follows^29^

2 $$\xi ={e}^{i\vartheta }{U}_{R}(\alpha ,\beta ,\tau ){\xi }_{0}={e}^{i\varphi ({\bf{r}})}(\begin{array}{c}{e}^{-i\alpha }{\cos }^{2}\frac{\beta }{2}\\ \sqrt{2}\,\cos \,\frac{\beta }{2}\,\sin \,\frac{\beta }{2}\\ {e}^{i\alpha }{\sin }^{2}\frac{\beta }{2}\end{array}),$$

where *ϑ* is a constant phase, *α*(**r**), *β*(**r**), and *τ*(**r**) are the spatial dependent Euler angles, *U*_*R*_(*α*,*β*,*τ*) = ${e}^{-i{F}_{z}\alpha }{e}^{-i{F}_{y}\beta }{e}^{-i{F}_{z}\tau }$ is the spin rotation operator, and *ϕ*(**r**) = *ϑ* − *τ*(**r**) denotes the phase of the condensate. As a result, the local spin can be expressed as **S**(**r**) = (sin *β* cos *α*, sin *β* sin *α*, cos *β*)^*T*^. Thus the order parameter manifold for the ferromagnetic phase is given by *SO*(3) and support three-dimensional Skyrmion, (which is also called as Shankar Skyrmion). As a three-dimensional Skyrmion, the spin texture is three-dimensional spatial dependent in BEC.

For the system (1), the Shankar Skyrmion is created through the action of magnetic field, in which the local spinor can be approximately expressed as

3 $$\xi ({\bf{r}},t)=\exp [\,-\,i{\omega }_{L}({\bf{r}})t\hat{{\bf{B}}}({\bf{r}})\cdot {\bf{F}}]{\xi }_{0},$$

which describes the Larmor precession of the spinor around the magnetic field, where *ω*_*L*_(**r**) = *μ*_*B*_ *g*_*F*_|**B**|/ℏ is the Larmor angular frequency, and $\hat{{\bf{B}}}$ = **B**/|**B**| denotes the unit vector of the magnetic field. Every point of the Shankar Skyrmion is realized by rotating the initial spinor *ξ*_0_ at position **r** through angle *ω*_*L*_*t* about the direction $\hat{{\bf{B}}}$. From Eq. (3), one can see that all atoms satisfy *ξ*(**r**, *t*) = *ξ*_0_ if |*ω*_*L*_(**r**)|*t* = 2 *lπ*, *l* = 1, 2, … is met, which establishes the boundaries of the Shankar Skyrmion indexed by *l* and any enclosed volume can therefore be compactified into the 3-sphere *S*^3^. The Three-Dimensional skyrmion is formed when any boundary approximately equals to the radius of the BEC. In the time interval, the skyrmion is not complete in the BEC but it will not decay in a short time. For the simplicity, we only consider the case *l* = 1. Thus, the Shankar Skyrmion can be classified by the third homotopy group *π*_3_(*SO*(3)) = ℤ and characterized by a topological number as follows^30^

4 $$Q=\frac{{m}^{2}}{16{\pi }^{2}{\hslash }^{2}}\,{\int }_{\Sigma }{{\bf{v}}}_{s}\cdot {{\boldsymbol{\Omega }}}_{s}{\rm{d}}{\bf{r}},$$

with ${{\bf{v}}}_{s}=i\hslash {\xi }^{\dagger }\nabla \xi /m$ and **Ω**_*s*_ = ∇ × **v**_*s*_ being the superfluid velocity and the vorticity, respectively. The topological number counts the number of times the space *SO*(3) is covered and can be calculated in a finite volume Σ:|*ω*_*L*_(**r**)|*t* ≤ 2*π* where the Skyrmion is restricted. Note that because spin vector field **S**(**r**) is in *S*^2^, we can expect a knot structure in the Shankar Skyrmion.

### Construction of magnetic filed

In our system, the magnetic field dominates the dynamics of the spin field. Three-dimensional quadrupole magnetic field with the form **B**(**r**) = *b*(*x*, *y*, −2*z*)^*T*^ can be used to create the three-dimensional Skyrmion with unit topological number in such system^24^. It is necessary to discuss the detailed structure of the quadrupole magnetic field. In the vertical direction, it is a gradient magnetic field with a zero-point at *z* = 0. In the horizontal plane, there is an isolated singular point at the origin (*B*_*x*_ = 0, *B*_*y*_ = 0). In the punctured plane ℝ^2^|∥{0}, we define the angle function *θ*_M_ of the two-dimensional magnetic field by

5 $${e}^{i{\theta }_{{\rm{M}}}}=\frac{{B}_{x}+i{B}_{y}}{\sqrt{{B}_{x}^{2}+{B}_{y}^{2}}},$$

which describes the change of the magnetic field direction in the horizontal plane. The total change in *θ*_M_ over a closed circle around the origin point divided by 2*π* is given by

6 $$W=\frac{1}{2\pi }\oint {\rm{d}}{\theta }_{{\rm{M}}},$$

and this turns out to be always a unit, independent of the radius of the circle. This integration define the winding number *W* of magnetic field around the closed circle.

**Figure 1:**
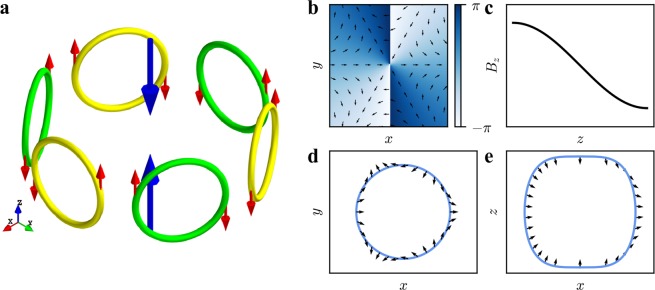
(**a**) The experimental set-up showing sextupole magnetic field generated by coils. The large blue arrows represent the laser beams. The small red arrows indicate the current directions. The configuration of the magnetic field **B**_2_ in the horizontal plane (**b**) and in the vertical direction (**c**), where the arrows and colors in (**b**) indicate the direction of the magnetic field and the scale of the angle function *θ*_M_, respectively. (**d**,**e**) The isolines of the magnetic field intensity in *xOy* plane and *xOz* plane, respectively, and the direction of the magnetic field at the isolines. Here, the parameters are *b*_2_ = 3.9 × 10^3^ G/cm^2^, *γ*_2_ = 200 *μ*m^2^ and *k* = 0.029 *μ*m^−1^.

Inspired by the fact, we here consider the horizontal multipole magnetic field **B**_mult_(**r**) = (*B*_*x*_, *B*_*y*_, 0), where the components *B*_*x*_ and *B*_*y*_ are given by

7 $${B}_{x}+i{B}_{y}={b}_{n}{(x-iy)}^{n}$$

with *n* being the positive integer. We can easily get the winding number *W* = −*n* from Eq. (6), where the negative sign means that the angle function *θ*_M_ decreases while traveling along the circle counterclockwise. The multipole magnetic field meets ∇ · **B**_mult_ = 0 and ∇ × **B**_mult_ = 0, and can be easily set by *n* + 1 pairs of Helmholtz coils (*n* is even) or anti-Helmholtz coils (*n* is odd) arranged around *z*-axis in experiment. A schematic plot for the case *n* = 2 can be found in Fig. 1(a).

In the vertical direction, a gradient magnetic field with a zero-point (*B*_*z*_∝*z*) should be still suitable for creating Skyrmions. Other functions, such as sin*z*, tan*z* and *z*^3^, which have the similar shape within certain range around the zero-point, can be considered as alternative solutions. But not to select *B*_*z*_∝*z*^2^, in this case, *B*_*z*_ ≥ 0 is always satisfied. Thus, the order parameter can only cover half of its manifold after rotating and therefore can not form a Skyrmion. Here we choose sinusoidal function as the magnetic field in vertical direction, i.e., *B*_*z*_∝ sin *z*, and the detailed mechanism for generating this kind of magnetic field is discussed below.

In order to construct the vertical magnetic field, we introduce an effective magnetic field induced from the interaction between light and atom, which can be characterized by **B**_eff_(**r**) = *ic*E^*^ × **E**, where the coefficient *c* depends on the details of the atomic structure as well as on the light frequency^31,32^. Such electric field may be realized in experiment. For the example, we can consider two laser beams polarized along $\hat{{\bf{x}}}$ and $\hat{{\bf{y}}}$, thus the corresponding electric field can be expressed by ${\bf{E}}=\sqrt{{\gamma }_{n}{b}_{n}/2c}{e}^{-i\Omega t}({e}^{-ikz}\hat{{\bf{x}}}+{e}^{ikz}\hat{{\bf{y}}})$, where *γ*_*n*_ is associated with the intensity of laser, Ω and *k* are the frequency and wavenumber of laser. The similar scheme has been used to produce an artificial spin-orbit coupling^33^. In this case, the effective magnetic field can be written as

8 $${{\bf{B}}}_{{\rm{eff}}}({\bf{r}})={b}_{n}{(0,0,-{\gamma }_{n}\sin (2kz))}^{T}.$$

Thus, we can construct a new magnetic field, which is superposition of the multipole magnetic field **B**_mult_(**r**) and the effective magnetic field **B**_eff_(**r**) as follows

9 $${{\bf{B}}}_{n}({\bf{r}})={{\bf{B}}}_{{\rm{mult}}}({\bf{r}})+{{\bf{B}}}_{{\rm{eff}}}({\bf{r}}).$$

Especially, when *n* = 2, the magnetic filed is the superposition of a sextupole field in the horizontal plane and a sinusoidal function in the vertical direction, which can be expressed as

10 $${{\bf{B}}}_{2}({\bf{r}})={b}_{2}{({x}^{2}-{y}^{2},-2xy,-{\gamma }_{2}\sin (2kz))}^{T}.$$

The detailed structure of the magnetic field given by Eq. (10) is shown in Fig. 1(b–e). From Fig. 1(b) one can see that, in *xOy* plane, the magnetic field exhibits a feature of the sextupole field and the angle function vary from −*π* to *π* twice along the clockwise around the origin, which means that its winding number equals to −2. Figure 1(c) presents the profile of the magnetic component *B*_*z*_, which is monotonous and possesses the feature of a gradient magnetic field with zero point at *z* = 0. In fact, the z-component of the magnetic field exhibits periodicity with z, and it can form a skyrmion in any half period. If the condensate is large enough, it will form skyrmion lattices. We consider the simplest case that the condensate only supports a single skyrmion. Figure 1(d,e) show the isolines of the magnetic field intensity in *xOy* and *xOz* planes, respectively, which are the closed curves. In the *xOy* plane, the magnetic field rotates two circles at the isoline, which similarly corresponds to the fact that the winding number is two. But in the *xOz* plane it only rotates semicycle. Comparing with the quadrupole magnetic field, the difference is that the winding number equals to 2. So, we suggest that the magnetic field can create a three-dimensional Skyrmion with topological number *Q* = 2.

### Creation of a three-dimensional Skyrmion

**Figure 2:**
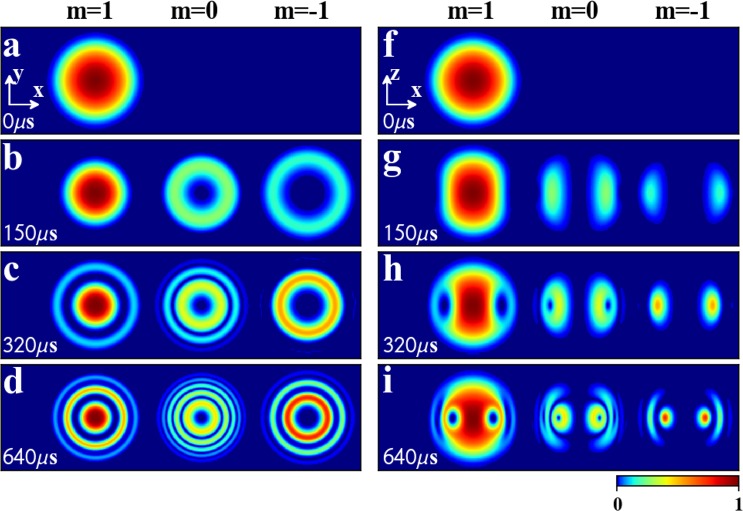
Detailed temporal evolution of density distributions of the order parameter during Skyrmion creation. The corresponding magnetic field **B**_2_ is described in Eq. (10). The left four columns are the cross section at *z* = 0 at (**a**) *t* = 0 *μ*s, (**b**) *t* = 150 *μ*s, (**c**) *t* = 320 *μ*s and (**d**) *t* = 640 *μ*s. The right four columns are the cross section at *y* = 0 at (**f**) *t* = 0 *μ*s, (**g**) *t* = 150 *μ*s, (**h**) *t* = 320 *μ*s and (**i**) *t* = 640 *μ*s. Here, the maximal density is normalized to unit and the field of view is 27.7 *μ*m × 27.7 *μ*m. The other parameters are the same as in Fig. 1.

We demonstrate the dynamics of BEC by numerically solving GP Eq. (1) in the magnetic field given by Eq. (10). In the numerical simulation, the initial state with spinor *ξ*_0_ can be obtained by calculating the ground state of the BEC in the presence of an uniform magnetic field in *z* direction, i.e., **B**_0_ = (0, 0, 0.1 G)^*T*^. In this case, all the atoms occupy in *m* = 1 state, i.e., spin up, as shown in Fig. 2(a,f). This is because the spin will be parallel with the magnetic field to minimize the energy in ferromagnetic state^29^. Then we change the uniform magnetic field to the magnetic field **B**_2_ and observe its evolution. Note that, based on the Thomas-Fermi approximation, the BEC in the harmonic potential is a ball-shaped distribution and its radius can be approximately determined by *R*_TF_ = [5*Nc*_0_/(4 *mω*^2^)]^1/5^. For our choice of the parameters, *R*_TF_ ≈ 11.4 *μ*m. Also, it can be shown that in the evolution the BEC is the rotational symmetric about *z*-axis. Note that the wavelength of laser is chosen to fit the shape of the BEC and the laser wavelength seems pretty long. However, in real experiment, if we use a pancake-shape BEC, thus a small laser wavelength is enough.

**Figure 3:**
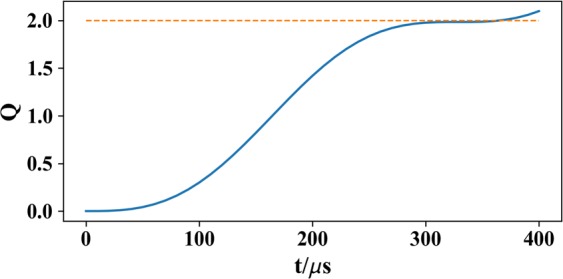
Time evolution of the topological number *Q* calculated in a spherical area with Thomas-Fermi radius *R*_TF_ ≈ 11.4 *μ*m. The corresponding magnetic field **B**_2_ is described in Eq. (10). The parameters are the same as in Fig. 1.

The density distributions at different evolution times are shown in Fig. 2. One can see that, atoms initially occupy in *m* = 1, as shown in Fig. 2(a,f). Under the action of the magnetic field **B**_2_, atoms gradually appear in *m* = 0 and *m* = −1, as shown in Fig. 2(b,g). Until *t* = 320 *μ*s, atoms in *m* = 1 form a distribution of a sphere surface, whose south and north poles are connected by a column. Atoms in *m* = 0 and *m* = −1 exhibit the profiles of a torus and a ring, respectively, as shown in Fig. 2(c,h). So a three-dimensional Skyrmion in spin field may be created in the BEC. Obviously, in the evolution, the system is in phase-separated and the sphere surface of *m* = 1 component forms the boundary of the Skyrmion. For such choice of the parameters, the boundary of the Skyrmion in spin field is determined by [(*x*^2^ + *y*^2^)^2^ + *γ*_2_^2^sin^2^(2*kz*)]^1/4^ = [2*π*ℏ/(*b*_2_*μ*_*B*_|*g*_*F*_|*t*)]^1/2^ ≈ 10.7 *μ*m, which equals approximately to *R*_TF_ given by the Thomas-Fermi approximation. Figure 3 presents time evolution of the topological number (4) and there is a plateau that the topological number *Q* ≈ 2 range from *t* = 300 *μ*s to 350 *μ*s. At *t* = 320 *μ*s, the numerical calculation for topological number within the boundary gives *Q* = 1.9960 ≈ 2. This integer-valued topological number confirms that the Skyrmion is created. With the further increasing of the evolution time, the Skyrmion will shrink but isn’t destroyed, as shown in Fig. 2(d,i). At the same time, the second ring in *m* = 1 and *m* = −1 appear, as shown in Fig. 2(d). According to Eq. (3), they correspond to |*ω*_*L*_(**r**)|*t* = 4*π* in *m* = 1, and |*ω*_*L*_(**r**)|*t* = 3*π* in *m* = −1, respectively, which are beyond the boundary of the Skyrmion given by |*ω*_*L*_(**r**)|*t* = 2*π*.

**Figure 4:**
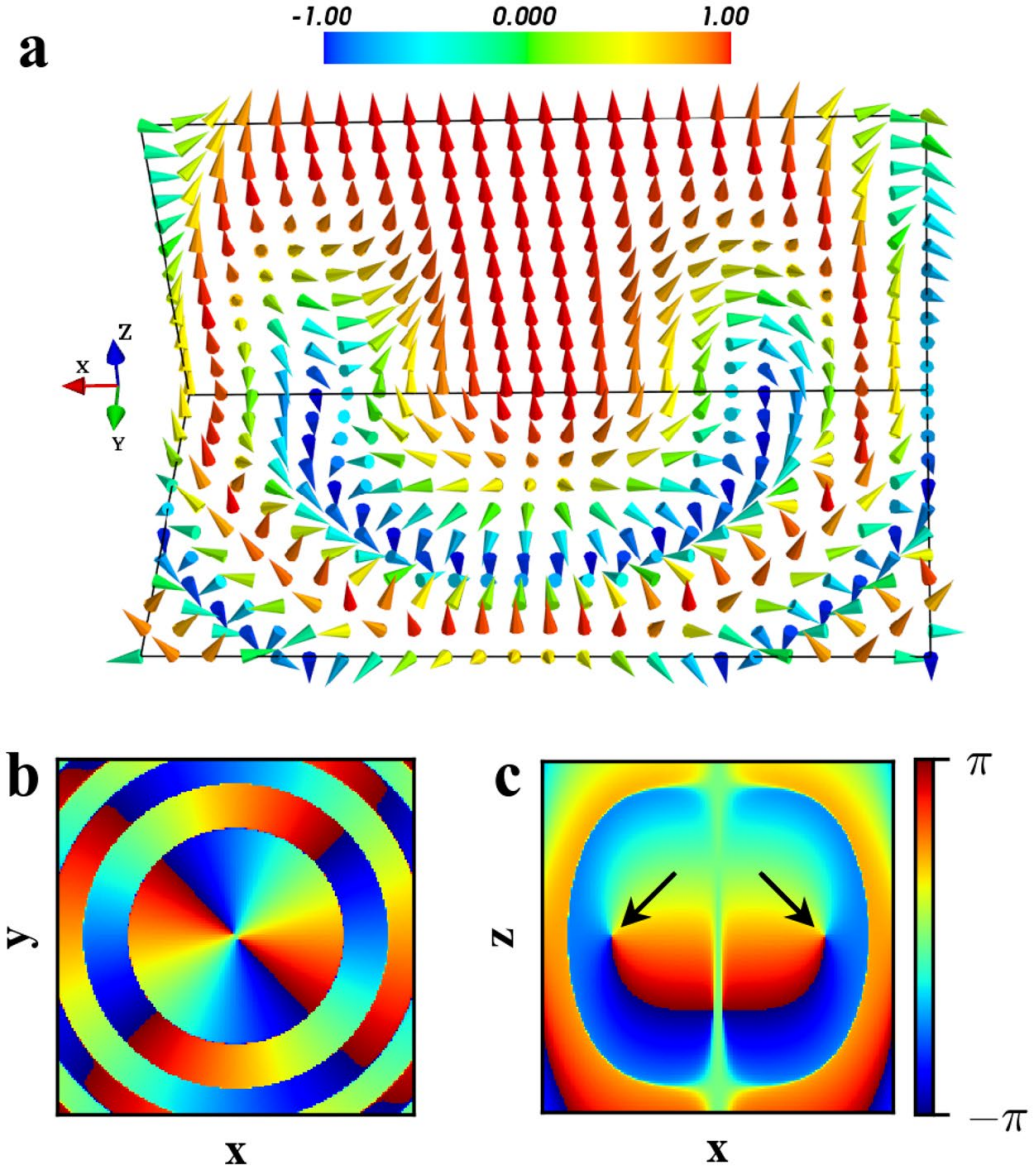
(**a**) The spin texture of **S** in the BEC created by the magnitic field **B**_2_ at *t* = 320 *μ*s, where the arrows and their colors indicate the spin direction and value of *S*_*z*_. (**b**,**c**) The distributions of the phase *ϕ*(**r**) for the order parameter in the *xOy* and *xOz* planes, where the arrows in (**c**) indicate the vortices. The parameters are the same as in Fig. 1.

To further understand the structures of the three-dimensional Skyrmion created by the magnetic field **B**_2_ at *t* = 320 *μ*s, Fig. 4 shows the spin texture of **S**(**r**) in the BEC and the distribution of the phase *ϕ*(**r**) for the order parameter. One can see from Fig. 4(a) that the spin field covered whole spin space (*S*^2^) in *xOz* plane, which can be reduced to be a two-dimensional Skyrmion. Also, from Fig. 4(b,c), one find that a vortex ring^23^ is formed in the BEC. In fact, in the *xOy* plane, as shown in Fig. 4(b), the distribution of the phase is separated into a disk with several rings isolated by the singular circles, which corresponds to *β* = 0,*π* from Eq. (2). So, the local spin at the singular circles points in turn downwards (*S*_*z*_ = −1) and upwards (*S*_*z*_ = 1), where the singular circle with *S*_*z*_ = −1 is the center of the vortex ring and the singular circle with *S*_*z*_ = 1 corresponds to the boundary of the Skyrmion. When *S*_*z*_ =  ± 1 (*β* = 0,*π*), the order parameter given by Eq. (2) as well as the spin vector is invariant under the transformations *ϕ* → *ϕ* + *π* and *α* → *α* + *π*. Hence, there is a *π* phase difference between the two sides of the singular circles. Obviously, the winding number of the phase in each ring equals to two. At the *xOz* plane, there are two vortices with the center located at the singular circle of *S*_*z*_ = −1, as shown by the arrows in Fig. 4(c). Therefore a vortex ring is formed along an axisymmetric singular circle in the BEC.

**Figure 5:**
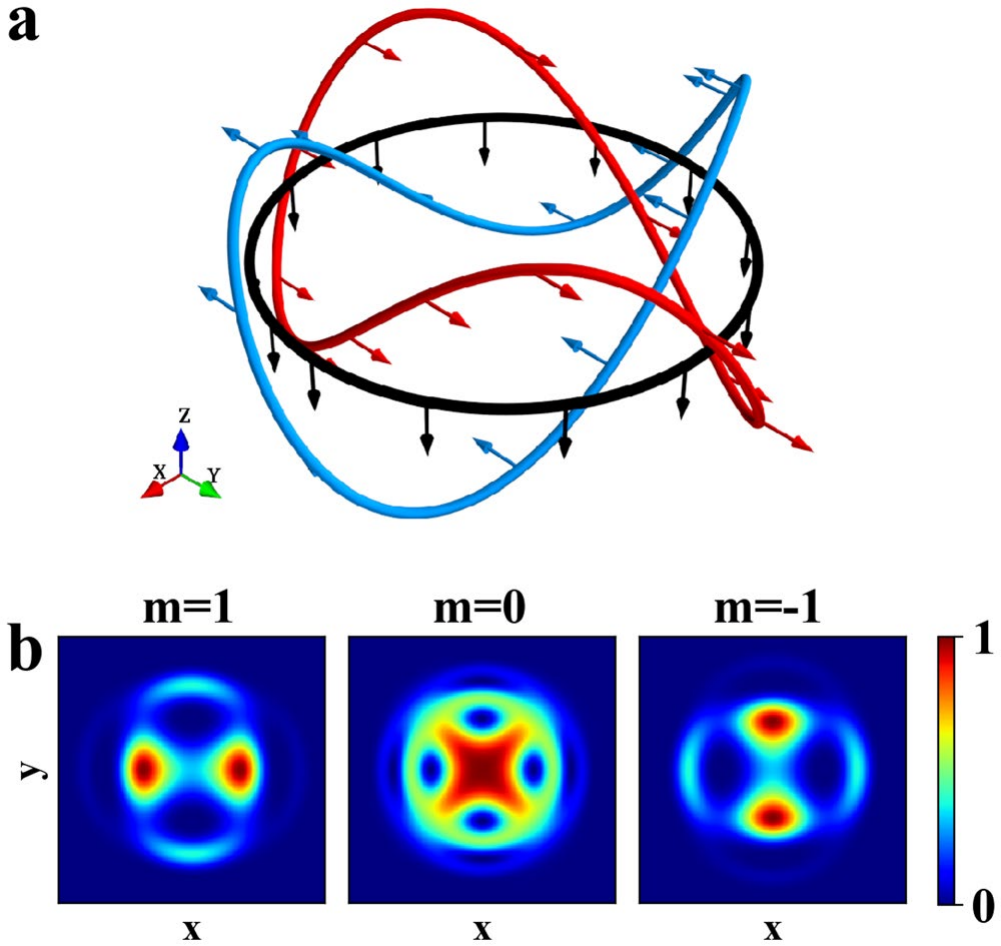
(**a**) The isolines of the spin field in the BEC created by the magnetic field **B**_2_ at *t* = 320 *μ*s, where the red, blue and black curves correspond to the cases of *S*_*y*_ = 1, *S*_*y*_ = −1 and *S*_*z*_ = −1, respectively. (**b**) The density distributions for three components of the order parameter in *xOy* plane with quantization axis along + *y*. The parameters are the same as in Fig. 1.

Figure 5(a) presents the isolines of the spin field in the BEC, where the red, blue and black curves correspond to the cases of *S*_*y*_ = 1, *S*_*y*_ = −1, and *S*_*z*_ = −1, and *S*_*z*_ = −1 is the core of the Skyrmion. One can see that these curves construct the fibers of Hopf fibration^34^, and each curve is linked with each other exactly twice so that any two curves consist of a solomon link, e.g., the red and blue curves. The property also exhibits the characteristics of the knot, and so create a knot-like Skyrmion in the spin field. In experiments, the three-dimensional Skyrmion can be described by the density distributions of the each component of the BEC with quantization axis along + *y*, which can be obtained by a unitary transformation, i.e., Ψ _+ *y*_ = *U*_+ *y*_Ψ with

11 $${U}_{+y}=(\begin{array}{ccc}-\frac{1}{2} & \frac{i}{\sqrt{2}} & \frac{1}{2}\\ \frac{1}{\sqrt{2}} & 0 & \frac{1}{\sqrt{2}}\\ -\frac{1}{2} & -\frac{i}{\sqrt{2}} & \frac{1}{2}\end{array}).$$

Figure 5(b) presents the density distributions for the order parameter in *xOy* plane with quantization axis along + *y*. One can see that the shape of *m* = 1 in the cross-section plane *z* = 0 appears as an ellipse and its major axis is along *y*-axis, which is corresponding to the red curve in Fig. 4(a). The similar pattern also appears in *m* = −1 while its major axis is along *x*-axis, which is corresponding to the blue curve in Fig. 4(a). The density distributions of *m* = 0 appears as a square within two circles and the outer circle is the boundary of the Skyrmion.

### Skyrmions with higher topological number

Now, we turn to discuss the more general case. First, we are going to reveal the topological connection between the magnetic field given by Eq. (9) and the Skyrmion. The three-dimensional Skyrmion is identified by a topological number of the map from real space *S*^3^ to the order parameter manifold *SO*(3), where the topological number is given by Eq. (4). Especially, when the spinor *ξ* is approximately replaced by Eq. (3), the topological number can be calculated analytically. In the following, we demonstrate the process under the action of the magnetic field given by Eq. (9).

Introducing the coordinate transformation

12 $$\{\begin{array}{c}x={(\rho \sin \theta )}^{\frac{1}{n}}\,\cos \,\phi ,\\ y=-\,{(\rho \sin \theta )}^{\frac{1}{n}}\,\sin \,\phi ,\\ -{\gamma }_{n}\,\sin (2kz)=\rho \,\cos \,\theta ,\end{array}$$

the unit vector of the magnetic field **B**_*n*_ can be expressed as ${\hat{{\bf{B}}}}_{n}={(\sin \theta \cos n\phi ,\sin \theta \sin n\phi ,\cos \theta )}^{T}$ and the magnitude can be expressed as |**B**_*n*_| = *b*_*n*_*ρ*, where *ρ*^2^ = (*x*^2^ + *y*^2^)^*n*^ + (*γ*_*n*_ sin(2 *kz*))^2^, *ϕ* varies from 2*π* to 0, *θ* varies from *π* to 0, and *ρ* aries from 0 to *ρ*_0_ = |2*π*ℏ/(*b*_*n*_*μ*_*B*_ *g*_*F*_*t*)|. The third formula in Eq. (12) imply *γ*_*n*_ ≥ *ρ*_0_ where *ρ*_0_^1/*n*^ is the boundary of the skyrmion. Therefore, the skyrmion is confined in a region along the z-direction and the condition −*π*/2 < 2 *kz* < *π*/2 is always satisfied. So, the spinor described by Eq. (3) can be expressed as

13 $$\xi =U\,\exp (i\lambda {F}_{z}){U}^{\dagger }{\xi }_{0},$$

where *λ* = −*ω*_*L*_*t* and *U* is an unitary operator in the form

$$U=(\begin{array}{ccc}{e}^{-2in\phi }{\cos }^{2}\frac{\theta }{2} & \frac{-1}{\sqrt{2}}{e}^{-2in\phi }\,\sin \,\theta  & {e}^{-2in\phi }{\sin }^{2}\frac{\theta }{2}\\ \frac{1}{\sqrt{2}}{e}^{-in\phi }\,\sin \,\theta  & {e}^{-in\phi }\,\cos \,\theta  & \frac{-1}{\sqrt{2}}{e}^{-in\phi }\,\sin \,\theta \\ {\sin }^{2}\frac{\theta }{2} & \frac{-1}{\sqrt{2}}\,\sin \,\theta  & {\cos }^{2}\frac{\theta }{2}\end{array}),$$

which can diagonalize the spin projection operator $\hat{{\bf{B}}}$ · **F** as *F*_*z*_, i.e., ${U}^{\dagger }\hat{{\bf{B}}}\cdot {\bf{F}}U={F}_{z}$. Employing Eq. (13), we can calculate the superfluid velocity

14 $$\frac{m{{\bf{v}}}_{s}}{\hslash }=\,\sin \,\theta \,\sin \,\lambda \nabla \theta +n\,{\sin }^{2}\,\theta (\cos \,\lambda -1)\nabla \phi -\,\cos \,\theta \,\nabla \lambda .$$

and the vorticity

15 $$\begin{array}{rcl}\frac{m{{\boldsymbol{\Omega }}}_{s}}{\hslash } & = & n\,{\sin }^{2}\theta \,\sin \,\lambda \nabla \phi \times \nabla \lambda \\  &  & +\,\sin \,\theta (\cos \,\lambda -1)\nabla \lambda \times \nabla \theta \\  &  & +\,2n\,\sin \,\theta \,\cos \,\theta (\cos \,\lambda -1)\nabla \theta \times \nabla \phi .\end{array}$$

Combining Eqs. (14) and (15), we find

16 $$\frac{{m}^{2}}{{\hslash }^{2}}{{\bf{v}}}_{s}\cdot {{\boldsymbol{\Omega }}}_{s}=2\,\sin \,\theta (1-\,\cos \,\lambda ).$$

Thus, the topological number *Q* given by Eq. (4) yields

17 $$Q=\frac{n}{8{\pi }^{2}}{\int }_{\pi }^{0}\,\sin \,\theta {\rm{d}}\theta {\int }_{2\pi }^{0}\,{\rm{d}}\phi {\int }_{0}^{2\pi }\,(1-\,\cos \,\lambda ){\rm{d}}\lambda =n.$$

Because the winding number for the multipole magnetic field **B**_mult_(**r**) given by Eq. (7) is *W* = −*n*, we can easily conclude that the absolute value of the winding number equals to the topological number of the corresponding Skyrmion, i.e., *Q* = |*W*|. Thus, we can create three-dimensional Skyrmion with arbitrary topological number theoretically.

**Figure 6:**
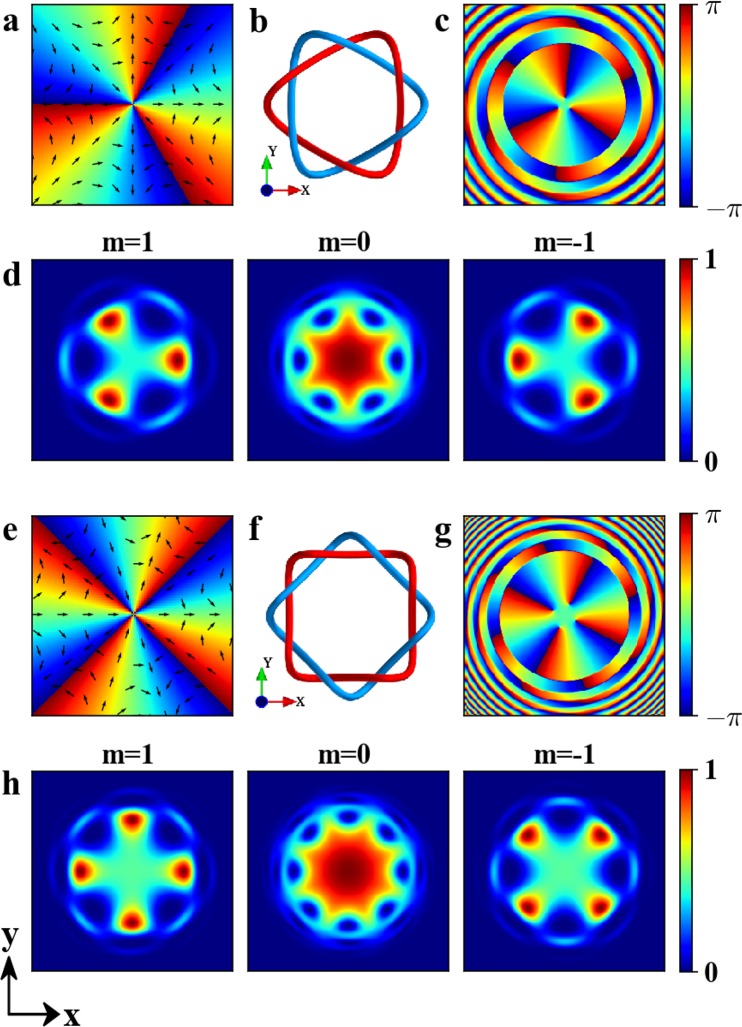
(**a**,**e**) The configurations of the octupole and ten-pole magnetic field, where the arrows and colors indicate the direction of the magnetic fields and the scale of the angle function *θ*_M_. (**b**–**d**) and (**f**–**h**) The structures of Skyrmions created by **B**_3_ and **B**_4_ at *t* = 320 *μ*s, respectively. Here, (**b**,**f**) the isolines of the spin vector, i.e., the red curve for *S*_*y*_ = 1 and the blue curve for *S*_*y*_ = −1. (**c**,**g**) The phase *ϕ*(**r**) of the order parameter in the *xOy*. (**d**,**h**) The density distributions for each components in *xOy* plane with quantization axis along +*y*. The maximal density is normalized to unit and the field of view is 27.7 *μ*m × 27.7 *μ*m.

As the examples, we will demonstrate the structures of the three-dimensional Skyrmions with the topological number *Q* = 3 and *Q* = 4 by applying octupole and ten-pole magnetic field. From Eq. (9), the detail magnetic field for *Q* = 3 is explicitly given by

18 $${{\bf{B}}}_{3}={b}_{3}{({x}^{3}-3x{y}^{2},-3{x}^{2}y+{y}^{3},-{\gamma }_{3}\sin (kz))}^{T},$$

where *b*_3_ = 3.7 × 10^−6^ G/*μ*m^3^, *γ*_3_ = 2.1 × 10^3^ *μ*m^3^ and the evolution time *t* = 320 *μ*s. Similarly, the detail magnetic field for *Q* = 4 is explicitly given by

19 $${{\bf{B}}}_{4}={b}_{4}{({x}^{4}-6{x}^{2}{y}^{2}+{y}^{4},4x{y}^{3}-4{x}^{3}y,-{\gamma }_{4}\sin (kz))}^{T},$$

where *b*_4_ = 3.4 × 10^−7^ G/*μ*m^4^, *γ*_4_ = 2.3 × 10^4^ *μ*m^4^ and the evolution time *t* = 320 *μ*s. The configurations and angle function of the octupole and ten-pole magnetic fields are shown in Fig. 6(a,e), respectively. From them, one can see that the magnetic fields exhibit the feature of the octupole and ten-pole field and the angle function vary from −*π* to *π* three times and four times along the clockwise around the origin, respectively, which means that their winding number is −3 and −4.

The numerical simulations of Skyrmions created by **B**_3_ and **B**_4_ are summarized in Fig. 6. In the Skyrmions, the red and blue curves, which are the isolines for *S*_*y*_ = 1 and *S*_*y*_ = −1, are linked exactly three times and four times, as shown in Fig. 6(b,f). The features of phase *ϕ*(**r**) for **B**_3_ and **B**_4_ are similar to Fig. 4(b) and the winding numbers are three and four, respectively, as shown in Fig. 6(c,g). The density of *m* = 0 component with quantization axis along +*y* exhibit a hexagram (see Fig. 6(d)) and octagram (see Fig. 6(h)) pattern, thus it is easy to distinguish Skyrmions with different topological number in experiment. The densities of *m* = 1 and *m* = −1 components correspond to the red and blue curves shown in Fig. 6(b,f), respectively. The numerical results also give the topological number *Q* = 2.9893 ≈ 3 for **B**_3_ and *Q* = 3.9751 ≈ 4 for **B**_4_.

## Discussion

In conclusion, we investigated the creation of a three-dimensional Skyrmion with topological number *Q* = 2 in spin-1 BEC by manipulating a sextupole magnetic field and a pair of counter-propagating laser beams. We described the structure of the Skyrmion by the spin vector and the phase of the order parameter. Meanwhile, the vortex ring and knot structure were found in the Skyrmion and were discussed in detail. The topological numbers of Skyrmions were calculated analytically and numerically in our model, which indicate that the method can be extended to create Skyrmions with arbitrary topological number. The numerical results confirmed that three-dimensional Skyrmions with *Q* = 3, 4 can be created. Thus, based on the recent experiment for create a Skyrmion with unit topological number^24^, we believe that this method can be extended to create Skyrmions with arbitrary topological number.

## Methods

Here we have investigated geometry properties of spinor Bose-Einstein condensates in different magnetic fields by numerically solving the mean field Gross-Pitaevskii equations. In detail, we use a norm-preserving imaginary time propagation method to solve the full three-dimensional Gross-Pitaevskii equations and employ a GPU to accelerate the numerical simulation. The topological numbers are analytically obtained, which also confirmed by the numerical results.
